# 

**Published:** 1856-10

**Authors:** 


### Contributors to Original Department of Vol. X.

JOSEPH RICHARDSON, D. D. S., M. D.
EDMUND OSMOND, D. D. S., M. D.
CHARLES BONSALL, D. D. S.
CHAS. H. ROBERTS, D. D. S.
AUTHOR OF "DENTAL WREATH."
C. B. CHAPMAN, A. M., M. D.
"A YANKEE."
SAMUEL MALLET, D. D. S.
H. R. SMITH, D. D. S., M. D.
DR. F. A. BREWER.
JAMES TAYLOR, D. D. S., M. D.
B, P. AYDELOTT, D. D., M. D.
E. COLLINS, D. D. S.
DR. L. P. HASKELL.
DR. W. MUIR ROGERS.
S. HUBBARD, M. D.
A. BERRY, D. D. S.
H. A. SMITH, D. D. S.
DR. H. M. SHEERAR.
J. TAFT.
GEO. WATT.

### SOCIETIES.

AMERICAN SOCIETY OF DENTAL SURGEONS.
AMERICAN DENTAL CONVENTION.
WESTERN DENTAL SOCIETY.
MISSISSIPPI VALLEY ASSOCIATION.
OHIO DENTAL COLLEGE ASSOCIATION.
ST. LOUIS DENTAL SOCIETY.

3375-37

### CONTENTS OF VOLUME X.

Action of Topical Remedies on Inflamed Dentine. By GEO. WATT, .................. 1
Alveolar Processes, Necrosis of. By J. RICHARDSON, D. D. S., M. D., .................. 16
American Society of Dental Surgeons. Sixteenth Annual Meeting, .................... 37
American Dental Convention. Second Annual Meeting of .......................... 63
An Improved Method of Using Gold Foil. By Prof. ROBERT ARTHUR, ................ 232
Amalgam. By J. D. WHITE, ................................................. 240
Alloying Gold. By G. WATT, ................................................ 396
Annealing Foil. By Dr. F. A. BREWER, ...................................... 400
Aluminium. By A. SNOWDEN PIGGOT, M. D., .................................. 440
Adhesive Gold Foil. By J. H. McQUILLEN, .................................... 448
Block or Cylinder Filling. By JAMES TAYLOR, ................................. 389
Chapman's Introductory Lecture, ............................................. 198
Classification of Decayed Cavities in the Teeth. By J. TAFT, .................... 230
Cylinder Filling. By JAMES TAYLOR, .......................................... 389
College of Dentists, England, ............................................... 476
Condensation of Gold Fillings. By W. M. ROGERS, ............................. 402
Dental Operations, ........................................................ 34
Dental Wreath. A Poem, ................................................... 153
Dental Chemistry of the Mouth. By G. WATT, ........................... 180, 305
Dental Convention, ........................................................ 215
Dental Periodicals. By JAMES TAYLOR, .................................225, 356
Digitalis, ................................................................ 276
Discussions of Mississippi Valley Association, ................................ 329
Editorial, .............................................................147, 256, 368 480
Essay upon Professional Fees. By E. TOWNSEND, D. D. S., M. D., ................. 169
Exciting Causes of Caries. By J. TAFT, ....................................... 187
Ergot and Digitalis in Hemorrhage, ......................................... 248
Excavators. By J. TAFT .................................................... 300
Functions of the Nervous System. By C. B. Chapman, A. M., M. D., ............ 277, 377
Filling Teeth, By R. ARTHUR, ............................................... 450
Gangrenous Degeneration of the Teeth and Gums, &c. By J. RICHARDSON, D. D. S., M. D., ................................................................. 16
Gutta Percha, as an Application to Clasps. By E. OSMOND, D. D. S., ............... 26
Hypertrophy of the Gums. By CHAS. BONSALL, D. D. S., ......................... 33
Impressions. By H. R. SMITH, D. D. S., M. D., ................................ 218
Improved Method of Using Gold Foil. By Prof. R. ARTHUR, ...................... 232
Inflamed Dentine. By H. A. SMITH, D. D. S., .................................. 383
Mode of Inserting Partial Sets. By Dr. F. A. BREWER, .......................... 224
Miscellaneous Items, ................................................ 252, 352 477
Models and Casts. By H. R. SMITH, .......................................... 272
Mississippi Valley Association—Minutes of .................................... 322
" " " Discussions of ........................................ 329
Minutes of Ohio Dental College Association, .................................. 346
Models and Castings, ...................................................... 403
Obituary, ................................................................ 152
One Idea, ................................................................ 195
Preparation of Plaster Models, .............................................. 32

Plate Forceps, .......... 217
Public Meeting of the Dental Profession, .......... 248
Professional Studies of the Dental Pupil. By B. P. Aydelott, D. D., M. D., .......... 310
Plates and Antagonizers. By H. R. Smith, .......... 405
Proceedings of Societies .......... 37, 322, 358, 412
Pluggers for Annealed Gold. By Louis Jack, D. D. S., .......... 445
Report of Western Dental Society, at Chicago, .......... 39
" " " " " St. Louis, .......... 412
Random Thoughts, .......... 192
Spirit Lamp Explosions, .......... 30
Simple Method of Overcoming a Difficulty, .......... 476
Sixteenth Annual Meeting of the American Society of Dental Surgeons, .......... 37
Second Annual Meeting of the American Dental Convention, .......... 63
Sensitive Dentine. By J. H. McQuillen, .......... 243
The Action of Topical Remedies on Inflamed Dentine. By Geo. Watt, .......... 1
The Exciting Causes of Caries By J. Taft, .......... 187
The Dental Profession and its Appropriate Work. By Geo. Watt, .......... 289
Thirteenth annual Meeting of the Mississippi Valley Association, .......... 322
Vascularity of Dentine. By J. Richardson, .......... 261
Wandering Thoughts. By H. M. Sheerar, .......... 401

TENTH VOLUME

OF THE

### DENTAL REGISTER OF THE WEST.

EDITED BY

J. TAFT and G. WATT.

TERMS: - - - - - - - - $3 PER ANNUM, IN ADVANCE.

### Advertisements Inserted as Follows:

| | |
|---|---|
| One page, per annum | $25 00 |
| One-half page, per annum | 15 00 |
| Quarter page, per annum | 10 00 |
| Card (five lines or less), | 5 00 |

Shorter periods than one year, by special arrangement.

☞Contributions to the pages of the *Register* respectfully solicited.

Exchanges and correspondents will please address, "Dental Register," or "Taft & Watt, Cincinnati, Ohio."

### CONTENTS.

### ORIGINAL ESSAYS AND COMMUNICATIONS.

The action of Topical Remedies on Inflamed Dentine. By Geo. Watt, ........ 1

Gangrenous Degeneration of the Cheek and Gums, with Necrosis and Exfoliation of the Alveolar Processes, and Maxillary Bone. By J. Richardson, D. D. S., M. D., ........ 16

Gutta Percha, as an application to Clasps. By Edmund Osmond, D. D. S., ........ 26

Spirit-Lamp Explosions, ........ 30

Preparation of Plaster Models, ........ 32

Hypertrophy of the Gums, ........ 33

Dental Operations, ........ 34

### PROCEEDINGS OF SOCIETIES.

Sixteenth Annual Meeting of the American Society of Dental Surgeons, ........ 37

Report of the Meeting of the Western Dental Society, at Chicago, Ill., in the International Hall, July 30th, 1856, ........ 39

Second Annual Meeting of the American Dental Convention, ........ 63

Editorial, ........ 147

Obituary, ........ 152

### BALTIMORE COLLEGE OF DENTAL SURGERY.

**SIXTEENTH ANNUAL SESSION, 1856-57.**

### FACULTY.

CHAPIN A. HARRIS, D. D. S., M. D.
*Principles of Dental Surgery.*

THOMAS E. BOND, M. D.
*Therapeutics and Materia Medica.*

WASHINGTON R. HANDY, M. D.
*Anatomy and Physiology.*

PHILIP H. AUSTEN, D. D. S., M. D.
*Mechanical Dentistry.*

EDWARD MAYNARD, D. D. S., M. D.
*Theory and Practice of Dental Surgery.*

REGINALD N. WRIGHT, M. D.
*Chemistry and Metallurgy.*

CHRISTOPHER JOHNSTON, M. D.
*Microscopical and Comparative Anatomy of the teeth.*

GEORGE W. NEIDICH D. D. S.
*Demonstrator of Dental Surgery.*

The Seventeenth Annual Session of the Baltimore College of Dental Surgery, will open on the first of October, and close on the third of March. The Regular Course of Lectures will commence on the third of November.

The month of October will be devoted to Practical Dentistry and Anatomical Dissections.

The large practice of the Infirmary offers to the Student superior facilities for the acquirement of a *practical* knowledge of Medical, Surgical, and Mechanical Dentistry.

Professors' Tickets, $110; Infirmary Ticket, $10; Dissection Ticket, (optional) $10; Matriculation Fee, $5; Diploma Fee, $30.

Good Board may be procured at from $3 50 to $4 00 per week. For further information address.

P. H. AUSTEN, *Dean of the Faculty.*

*No.* 76 *Sharp Street.*

---

### DENTAL DEPOT.

### JOHN KLEIN,

MANUFACTURER OF

### PORCELAIN TEETH,

### Gold & Tin Foil. Gold & Silver Plate, Wire, Spiral Springs

INSTRUMENTS, &c., &c.

*No.* 16½ *North Eighth street, Philadelphia, Pa.*

Constantly on hand a general assortment of Improved Plate, Pivot, Molar, Bicuspids, and Gum Teeth, at reduced prices; together with an assortment of Dental Instruments, Materials, Tools. Lathes, Wheels, Molding Flasks, Gold and Tin Foils, Files, Blow Pipes, Lamps, Head Rests, &c., &c. All Orders promptly filled.

